# Evaluation of the Effect of Storage Time on ROTEM S^®^ Parameters in Healthy and Ill Dogs

**DOI:** 10.3390/ani12151996

**Published:** 2022-08-07

**Authors:** Nicole Weingand, Johanna Vuille-dit-Bille, Rahel Jud Schefer, Annette P. N. Kutter, Martina Stirn, Katja-Nicole Adamik, Nadja E. Sigrist

**Affiliations:** 1 Department for Small Animals, Vetsuisse Faculty, University of Zurich, 8057 Zurich, Switzerland; 2 Department of Clinical Veterinary Science, Vetsuisse Faculty, University of Bern, 3012 Bern, Switzerland; 3 Department for Clinical Diagnostics and Services, Vetsuisse Faculty, University of Zurich, 8057 Zurich, Switzerland

**Keywords:** thromboelastometry, canine, reference interval, Ex-TEM, In-TEM, Fib-TEM

## Abstract

**Simple Summary:**

Bleeding disorders can cause life-threatening illness in dogs. The need for fast recognition and diagnosis of these conditions is therefore of the utmost importance to have a positive impact on the patients’ survival. In the past decade, the use of viscoelastic testing for rapid assessment of global haemostasis has gained popularity. However, the most reliable time for testing after blood collection has not been determined. For this reason, blood samples were taken from healthy client-/staff-owned dogs and repeated measurements were performed at three different time points (10 min, 30 min, and 70 min after blood collection). Additionally, a group of currently ill patients was included and Ex-TEM S measurements were performed at the same three timepoints. We found that there was a significant change of results over time, suggesting the need for time-specific reference intervals. Which of these time points reflects the “true” coagulation status of our patients currently remains unknown.

**Abstract:**

Viscoelastic testing as a bedside test to assess global haemostasis has gained popularity in the past decade, with rotational thromboelastometry (ROTEM) and thromboelastography (TEG) being the two commonly used devices. TEG studies suggest analysis 30 min after blood sampling. However, the reproducibility of results over time for ROTEM analysis using lyophilized samples in dogs has not been established. In this study, we investigated the influence of time on viscoelastic testing, using 33 healthy staff-/client-owned dogs for blood sampling and repeated measurements of ROTEM tracings at three different time points after blood collection. Additionally, a group of 21 hospitalized patients with suspected coagulation disorders were included to investigate whether stability over time was comparable between healthy and ill dogs. We demonstrated a significant difference of ROTEM tracings over time, with a tendency towards hypocoagulability over time. These changes do have a clinical relevance as they exceed reference intervals and could therefore lead to erroneous conclusions about a patient’s coagulation status. Therefore, time-specific reference intervals are proposed and presented in this publication.

## 1. Introduction

Coagulation disturbances may cause life-threatening illness and rapid diagnosis of the underlying pathology of an acute coagulation disorder is mandatory.

The use of viscoelastic measurements for detecting coagulation disorders in veterinary medicine gained popularity within the past decade [1,2,3,4]. Viscoelastic coagulation analysis allows for evaluation of haemostasis in whole blood from initiation of a fibrin clot to its maximum strength and the dissolution of the clot [5]. The two commonly used viscoelastic tests are thromboelastography (TEG) and rotational thromboelastometry (ROTEM). Rotational thromboelastometry analysis is performed with a fixed cup and an oscillating pin. While the pin rotates within the blood sample, its movement becomes restricted by clot formation and later liberated again by its lysis. Therefore, it provides information on clot formation kinetics and strength [6]. A real-time graph (temogram) is displayed on a screen and first conclusions can be made within 5–10 min [7]. 

Because of the quick availability of first results [8,9,10,11,12,13], the use of ROTEM in emergency settings is of great interest. However, fast results require immediate analysis after blood sampling. While the manufacturer recommends storage at 37 °C and immediate analysis in people, the Partnership of Rotational ViscoElastic Test Standardization (PROVETS) guidelines recommend a storage time of 30 min at room temperature [14]. Studies in people analysing the influence of storage time on coagulation parameter results show stable results for ROTEM parameters analysed between 0–120 min after blood collection of healthy volunteers [15,16]. However, a study investigating coagulability of coagulopathic trauma patients found a significant change in ROTEM tracings 0–60 min after blood sampling [17].

In veterinary science, studies about storage time and sample technique exist only for TEG [18,19] and the conclusions from these studies have been extrapolated to ROTEM analysis [20]. The optimal storage time of blood prior to ROTEM analysis has not been determined. A single study investigating weak and strong tissue factor activation reports that results measured 0 and 30 min after withdrawal are not different if a strong clotting activator is used. A weak activator led to hypercoagulability within the first 30 min of storage time [21]. According to the manufacturer, ROTEM S tests contain activators that lead to moderate activation of coagulation. Based on PROVETS guidelines, reference intervals for ROTEM parameters have been determined approximately 30 min after blood sampling [22,23] and it is currently unknown if earlier (for example in an emergency patient) or later (in research settings) analysis has an influence on results. Specifically, patients with acute bleeding disorders would benefit from immediate analysis and therefore faster availability of results. 

The aim of this study was to determine whether the time of ROTEM analysis after blood sampling has an influence on results. The null hypothesis was that samples analysed 10 or 70 min after blood sampling were not significantly different from samples analysed 30 min after blood sampling. A second aim was to determine if coagulation status (hypo-, normo- or hypercoagulable) and sampling technique (jugular venipuncture using a syringe vs. free-flowing blood collection from a saphenous vein) influences consistency of results over time.

In case of significant differences at the measurement points, an additional goal was to determine reference intervals for measurements after 10 and 70 min, respectively.

## 2. Materials and Methods

The study was conducted at the Small Animal Clinic of the Vetsuisse Faculty, University of Zurich between March and May 2021 and was completed together with another study investigating pre-analytical factors that could influence test results [24]. It was approved by the ethics committee on animal research of the Canton of Zurich (ZH 057/19) and owner consent was obtained. Sample size was chosen based on previous studies [16,21].

Student-, staff-, and client-owned dogs were recruited for blood sampling. Demographic data (age, breed, sex, and body weight), current medication, and medical history were recorded of all dogs. Chronic illness or current medication were not an exclusion criteria. Dogs were excluded when they weighed < 2 kg, were younger than 10 months, or were too stressed for blood sampling. 

### 2.1. Blood Sampling

Blood sampling was performed on one or two phlebotomy sites (vena *jugularis* and/or vena *saphena lateralis*) depending on the weight (two phlebotomies were only performed if bodyweight > 10 kg), the character of the dog, and the owner’s consent. 

For dogs with one phlebotomy site, either the jugular vein or the lateral saphenous vein was prepared aseptically and approximately 4 mL blood was collected. For dogs with two phlebotomy sites, both the jugular vein and the lateral saphenous vein were prepared aseptically, and blood was drawn first at the jugular vein (3 mL) followed immediately by blood collection at the lateral saphenous vein (2.6 mL). 

Standardized venipuncture was performed by two operators (JV and NW) to reduce the influence of preanalytical errors [14,20,25]. For jugular vein sampling, a 22 G needle was connected to a 5 mL syringe and blood was drawn using minimal vessel occlusion and mild aspiration with a vacuum of 1 ml inside the syringe. After needle removal, the blood was filled into two or three 1.3 ml 3.8% sodium citrate tubes (SAB500 Sarstedt blood collection tube, 1.3 mL, 3.2% sodium citrate) with a strict 1:9 ratio. Blood sampling at the lateral saphenous vein was performed with a 22G needle and blood was collected free-flowing in two or three 1.3 mL 3.8% sodium citrate tubes with a strict 1:9 ratio. Each tube was inverted carefully several times and was then placed on the analysers’ warming plate. 

### 2.2. ROTEM Analysis

Viscoelastic testing was performed by three trained operators on two ROTEM devices with 4 channels each (TEM innovations GmbH, Munich, Germany) with one operator handling one device at a time. ROTEM analysis was performed using single test vials for extrinsic rotational thromboelastometry—Ex-TEM S (tissue factor-activated temogram), intrinsic rotational thromboelastometry—In-TEM S (ellagic acid-activated temogram) and fibrinogen rotational thromboelastometry—Fib-TEM S (tissue factor-activated temogram with platelet inhibition) (all TEM innovations GmbH, Munich, Germany). From each tube, three serial measurements of the same test were performed (e.g., of the three tubes, one was used for Ex-TEM S, one for In-TEM S, and one for Fib-TEM S analysis). Each tube was rested at 37 °C for at least 3–5 min prior to the first analysis. The first ROTEM measurement was performed 10 +/− 2 min, the second measurement 30 +/− 2 min and the third measurement 70–80 min after blood sampling. The channels were chosen randomly by the tester; however, the first and the third measurement were performed at the same channel to exclude channel-dependent variability. If there were two sampling sites, only two tests could be performed due to channel availability. For each sample, an Ex-TEM S was performed. The second test was chosen randomly, with the aim of having a similar number of In-TEM S and Fib-TEM S results.

Samples were analysed according to the manufacturer instructions. Briefly, the cups and pins were placed correctly, and the reagents were allowed to reach room temperature, by placing them in the designated spaces on the device approximately 10 min prior to analysis. Using an automated pipetting program provided by the device, 300 µL of 37.0 °C warm, citrated whole blood was incubated for 5 s with the appropriate single portion reagent (Ex-TEM S, Fib-TEM S, In-TEM S) and afterwards pipetted into the ROTEM cuvette (Cup and Pin Pro, TEM Innovations GmbH, Munich, Germany). The cuvette was then connected to the pin and the measurement was started.

The running time of all samples was 60 min with the exemption of Fib-TEM S tracings, which were stopped after 30 min if an additional channel was needed. Every temogram was visually evaluated for artefacts by two of the investigators (N.E.S., N.W.). The following parameters were further analysed: clotting time (CT—time from start of the measurement until the initiation of a clot with an amplitude of 2 mm); clot formation time (CFT—time between 2 mm and the time until the clot reached an amplitude of 20 mm); α-angle (α—angle between baseline and clotting curve; going through the CT point); maximum clot firmness (MCF—maximum amplitude reached in the measurement); maximum clot elasticity (MCE—a parameter for the clots’ elasticity calculated as E = 100 × MCF/(100−A)); maximum lysis (ML—difference between MCF and the lowest amplitude after MCF is the maximum lysis detected during the runtime); amplitude at 10 min (A10—the amplitude reached by the clot 10 min after test start); and G (Shear Elastic Modulus Strength, which is calculated as 5000 × MCF/(100−MCF)).

### 2.3. Additional Blood Analysis

A venous blood gas analysis was performed by placing 0.3 mL of the left-over blood into a heparinized syringe (BD A-line blood gas syringe, Becton Dickinson and Company, Plymouth UK) immediately followed by analysis on a point-of-care blood gas analyser (RAPID Point 500, Siemens Healthcare, Zurich, Switzerland). 

Micro-haematocrit, total solids and a blood smear for manual thrombocyte counting was performed from left-over blood of one of the citrate tubes of each blood sampling localization. Serum colour was noted. Blood smears were stained using a Wright-Giemsa stain (Diff-Quick, Medion Diagnostics, Düdingen, Switzerland) and thrombocyte number was estimated by counting the average number of thrombocytes seen in 100× oil immersion (Olympus CX 43, Olympus Europa SE & Co. KG, 20097 Hamburg, Germany). A total of 10 monolayer high-power fields were viewed, and the average platelet count was multiplied by 15.000. All blood smears were interpreted by the same operator (N.W.).

### 2.4. Clinical Cases

Additionally, leftover citrate blood from patients undergoing ROTEM analysis for clinical purposes were included if the first ROTEM analysis was performed 10 +/− 2 min after blood sampling. Since there was generally only one citrate tube available, only Ex-TEM at the 30 and/or 70 min timepoint was repeated. 

### 2.5. Coagulation Status

Based on previously established reference intervals for G [22], a calculated parameter considered to be a measure of complete clot strength, tracings were categorized as hypocoagulable, hypercoagulable or normocoagulable [26]. G at 30 min was considered as reference coagulation status. 

Study dogs were deemed healthy based on a physical exam, history, haematocrit, electrolytes, blood gas, glucose, and lactate analysis (RAPID Point 500, Siemens Healthcare, Zurich, Switzerland) were used for determination of reference intervals at the 10 and 70 min timepoints.

### 2.6. Data Analysis and Statistics

Data from the ROTEM database was copied into an excel sheet. The database was manually checked for errors by two authors (NW, NS). Statistical analysis was performed using IBM SPSS Statistics version 27 (IBM Corporation, Armonk, New York, NY, USA).

All parameters were tested for normality in their distribution using the Shapiro–Wilk test. Afterwards, data were tested for outliers using Tukey analysis. No dataset was excluded from further analysis. Because most of the measured values were not normally distributed, for all further statistical tests non-parametrical tests were chosen and results are displayed as median and range (min–max). Friedman and Wilcoxon rank tests were performed to analyse changes over time (repeated measurements) and post hoc correction was performed using Bonferroni correction.

Multiple logistic regression for change in coagulation status between T10–T30 and T30–T70 was performed with the covariant factors platelet number, haematocrit, haemolysis (yes/no), and sampling site (jugular/peripheral).

A 95% confidence interval was set and a *p*-value < 0.05 was set as statistically significant.

Reference intervals at T 10 and T 70 were performed from 36 clinically healthy dogs without current medication using a statistical program (MedCalc^®^ Statistical Software version 20.027 (MedCalc Software Ltd., Ostend, Belgium; https://www.medcalc.org; accessed on 16 March 2022)

Reference intervals were reported as 2.5th–97th percentile with 90% confidence interval were determined by a nonparametric method (non-parametric data) or following CLSI guidelines for percentiles and their confidence interval (normally distributed data). 

## 3. Results

A total of 55 dogs were enrolled. One dog was excluded as the dog’s blood clotted inside the tubes. The 54 included dogs were aged between 9 and 200 months (median, 82 months) and had a body weight ranging from 2.3 to 59 kg (median, 21.6 kg). In total, 11 (20.4%) were intact males, 18 (33.3%) castrated males, 7 (13.0%) intact females, and 18 (33.3%) castrated females. Of these, 16 were mixed-breed dogs and 38 were pedigree dogs; 5 of them were Labrador Retrievers, 3 Border Collies, and 3 French Bulldogs; there were 2 from each of the following breeds—German Shepherd, Pit Bull Terrier, German Wirehaired Pointer, Golden Retriever, Chihuahua, Australian Shepherd, Akita Inu; and there were 1 from each of the following breeds—Rhodesian Ridgeback, Poodle, Bolonka Zwetna, Magyar Vizsla, Malinois, Greater Swiss Mountain Dog, Schappendoes, Whippet, Papillon, Labradoodle, Maltese, Berger Blanc Suisse, and Dachshund. 

The haematocrit ranged from 25 to 52% (median, 40%, reference interval 36–54%) and 9 dogs showed haematocrits below the reference interval. Total solids ranged from 41 to 78 g/L (median, 59 g/L, reference interval 53–76 g/L) and thrombocyte count ranged from 0/µL to 587,000/µL (median, 200,000/µL, reference interval 150–399,000/µL). Ionized calcium measurements were available in 20 dogs and ranged from 1.18 to 1.36 mmol/L (median, 1.28 mmol/L; reference interval 1.25–1.4 mmol/L).

A total of 21 of 54 dogs (39%) were client-owned dogs, which presented with different suspected or proven bleeding disorders including haemoabdomen (n = 3), immune-mediated thrombocytopenia (n = 3), and *Angiostrongylus vasorum* infection (n = 2), among other diagnoses. 

### 3.1. ROTEM S Parameter Changes over Time

A total of 71 blood samples, 50 from 33 staff-/student-owned dogs and 21 from patients, were analysed. Ex-TEM S and In-TEM S analysis was performed at all investigated timepoints (T10, T30, T70) in 68/71 and 35/71 samples, respectively, while Fib-TEM S analysis was performed in 28/71 blood samples.

**Figure 1:**
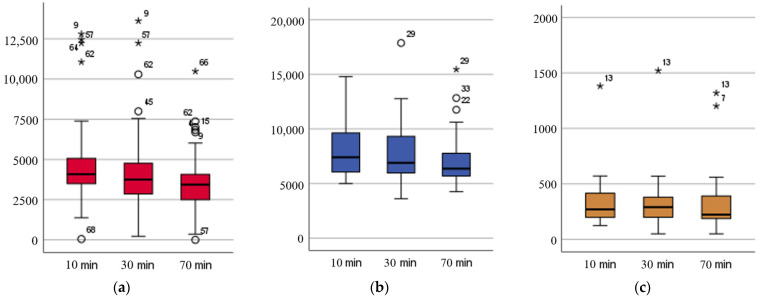
Box-plot graphs of G-value analysed 10, 30, and 70 min after blood sampling (**a**) Ex-TEM S (**b**) In-TEM S, (**c**) Fib-TEM S.

Table 1 summarizes median values of the investigated parameters at each timepoint. Ex-TEM S analysis showed a significant change over time in all investigated parameters (*p* < 0.01), except from CT (*p* > 0.05) (Figure 1). With the exemption of CT and ML, all In-TEM S parameters evaluated showed significant changes between T10 and T70 and T30 and T70, but not T10 and T30 (*p* < 0.05). For Fib-TEM tracings significant changes were only observed between T10–T70 (*p* < 0.05) (Table 1). Changes in G-value over time are shown in Figure 1. 

### 3.2. Parameter Changes over Time Based on Coagulation Status

Based on G at the timepoint T30, 51/68 (75%) Ex-TEM S tracings, 27/35 (77%) In-TEM S tracings and 23/28 (82%) Fib-TEM S tracings were classified as normocoagulable; 11/68 (16%) Ex-TEM S, 6/35 (17%) In-TEM S and 2/28 (7%) Fib-TEM S as hypocoagulable; while 6/68 (9%) Ex-TEM S, 2/35 (6%) In-TEM S and 3/28 (11%) Fib-TEM S tracings were hypercoagulable. 

Parameter changes over time are summarized in Table 2, Table 3 and Table 4.

For normocoagulable samples, the results were very similar to those of the whole population (Table 2). All investigated Ex-TEM S variables significantly (*p* < 0.05) differed between time points, except from CT at all timepoints (*p* > 0.05), and α-angle between T10 and T30 (*p* > 0.05).

The results for normocoagulable In-TEM S parameters matched the whole population group, showing significant changes over time only between T10 and T70 (*p* < 0.001) and T30 and T70 (*p* < 0.001) There was no significant difference for CT and ML (*p* > 0.05) between any of the timepoints (Table 2).

In normocoagulable Fib-TEM S tracings, significant changes over time were observed between T10 and T70 for A10, MCF, and MCE (all *p* < 0.05) (Table 2).

In hypocoagulable Ex-TEM S samples (Table 3), significant changes between the timepoints T10 and T30 were noted for A10, CFT, MCF, MCE and G (all *p* < 0.05), while in hypocoagulable In-TEM S tracings, MCF, MCE, and G changed significantly between T10 and T30 (all *p* < 0.05). No significant changes could be observed between T30 and T70. 

For hypercoagulable Ex-TEM S samples significant changes between T30 and T70 were found for CFT, MCE, and G (all *p* < 0.05) (Table 4). Hypercoagulability was only recognized in 2 In-TEM S and 3 Fib-TEM S samples and changes over time were not analysed.

### 3.3. Change of Coagulation Status

With Ex-TEM S analysis, 7/70 (10%) of hypercoagulable samples at T10 turned to normocoagulability at T30, and 5/70 (7.1%) of normocoagulable samples at T10 were interpreted as hypocoagulable at T30 (Table 5). 

Ex-TEM—tissue factor activated temogram; In-TEM—allegic acid-activated temogram; Fib-TEM— tissue factor-activated temogram with platelet inhibition; T10—10 minutes after blood sampling; T10—10 min after blood sampling; T30—30 min after blood sampling; T70—70–80 min after blood sampling. Significant *p*-values are presented in bold.

Between T30 and T70, 3/68 (4.4%) of samples changed from hypo- to normocoagulability and another 3/68 (4.4%) from normo- to hypocoagulability, 1/68 (1.5%) turned from hypercoagulable to normocoagulable and 1/68 (1.5%) changed from hyper- to hypocoagulable (Table 5) in comparison with T 30 tracings.

In In-TEM S tracings, 4/35 (11.4%) of normocoagulable objects at T10 turned hypocoagulable at T 30 and 1/35 (2.9%) became hypercoagulable. In the time between T30 and T70 1/35 (2.9%) sample changed from hypocoagulability to normocoagulability (Table 5).

In Fib-TEM S tracings, 2/28 (7.1%) normocoagulable tracings at T10 changed to hypocoagulable tracings at T30. One of 28 (3.6%) samples changed from hyper- to normocoagulability and another 1/28 (3.6%) turned from normo- to hypercoagulability. At T70 2/28 (7.1%) samples turned from normo- to hypercoagulability, whereas 1/28 (3.6%) turned from hyper- to normocoagulability (Table 5) when compared to T30 tracings.

### 3.4. Effect of Sampling Site, Degree of Haemolysis, Haematocrit, and Platelet Count

Binary logistic regression showed that haematocrit was a significant covariant for a change in Ex-TEM S coagulation status between T10–T30 but not T30–T70. The probability of a coagulation status change in Ex-TEM S decreased by 20% with each percent increase in haematocrit (n = 50, *p* = 0.035, R2 = 0.386, Cohens f2 = 0.63, odds ratio 0.802, 95% confidence interval 0.652–0.986). None of the analysed covariants (haematocrit, platelet number, haemolysis, sampling site) were significantly associated with a change in In-TEM S or Fib-TEM S coagulation status. 

### 3.5. Reference Intervals for Analysis 10, 30, and 70 min after Blood Sampling

Reference intervals for Ex-TEM S, In-TEM S, and Fib-TEM S parameters at 10 and 70 min after blood sampling are summarized in Table 6. Most reference intervals significantly differed over time (Table 6).

## 4. Discussion

Repeated analysis of ROTEM S parameters measured in citrated canine whole blood showed significant changes over time. These changes were identified in all evaluated ROTEM S tests (Ex-TEM, In-TEM, Fib-TEM), with more affected parameters in Ex-TEM S tracings compared to In-TEM S or Fib-TEM S analysis. Additionally, the changes had a significant effect on the interpretation of coagulation status based on available reference intervals. 

These findings are in contrast with equivalent studies in people, where good stability over time was reported (CV < 6% in all assays) [27]. Although some significant changes were identified in Fib-TEM and In-TEM analysis when different devices were used in people, none of these changes had any therapeutic consequence because changes were within the reference range [16,27]. 

When classifying the coagulation status of a ROTEM tracing based on the G value, our results showed a clinically relevant change over time. Specifically, some tracings defined as hypocoagulable at T30 were interpreted as normocoagulable at T10 when using the institution’s reference interval evaluated 25–35 min after blood sampling. Therefore, new reference intervals for analyses after 10 min and 70 min were generated. These new reference intervals, despite being based on low numbers of patients, significantly differed between different time points. 

An earlier study in dogs investigating storage time and activator use in ROTEM analysis showed a trend towards hypercoagulability over time. However, these changes were minor as long as a strong activator was used [21]. According to the manufacturer, ROTEM S tests contain activators that lead to moderate activation of coagulation, indicating that, if at all, a trend towards hypercoagulability over time could be expected. 

Studies evaluating the influence of time on ROTEM analysis in people show contradictory results. In healthy people, ROTEM parameters were shown to be stable over 120 min [16]. A study evaluating blood from trauma patients showed a spontaneous improvement in clot firmness with time [17]. Since these changes were not identified in Fib-TEM tracings, this finding was suspected to be caused by a change in platelet function following trauma. Thromboelastography studies in healthy adults also showed a trend towards hypercoagulability over time [15,28]. In these TEG studies, results were more hypocoagulable immediately after recalcification; this finding was explained by delayed thrombin formation in recalcified samples [15,28], leading to the recommendation of 30 min storage time before analysis.

These results from TEG and ROTEM stands in contrast with our results showing a trend for tracings becoming more hypocoagulable over time. The reasons for changes over time in ROTEM parameters and specifically the trend towards hypocoagulability over time has not been described so far and need further investigation. A possible explanation may be the complex method of platelet activation. During normal conditions, platelets flow in the blood stream without interacting with the endothelial surface or each other. However, platelets are very reactive to external stimuli and endothelial damage [29]. As soon as thrombocyte activation starts, the formation of a reversible clot is initiated. This clot relies on an irreversible activation of αIIbβ3 for stable clot formation [30,31]. Therefore, platelet activation and aggregation to each other induced by blood sampling could explain the hypercoagulable tendency found at T 10 while after 30 min, platelet activation has resolved. Of note, all previous studies have investigated liquid ROTEM reagents, which, in contrast to the lyophilized ROTEM S reagents in our study, require recalcification of the reagent prior to analysis. A difference in reference intervals between multi-test vials and single-test vials has been found in a previous study of [22]. The reason for this discrepancy is unclear; however, an insufficient activation of canine blood by tissue factor has been discussed [22].

Ex-TEM S was the most sensitive to changes over time. Tissue factor, the activator used in Ex-TEM S, and ellagic acid (In-TEM S) are considered strong activators [14,21]. Previous studies in dogs investigating lyophilized ROTEM reagents suspected that tissue factor concentration in Ex-TEM S and Fib-TEM S reagents may be insufficient for complete activation of coagulation in canine blood, leading to smaller MCF reference intervals compared to reference intervals determined with liquid Ex-TEM reagents [22]. The clot formation 10 min after blood sampling may then be improved due to platelet activation as described above, while measurement 30 min after blood sampling shows longer CT and CFT.

Preanalytical factors that may influence results of viscoelastic testing include sampling technique [19], venipuncture quality [32], haemolysis [33], and haematocrit [34] with hypercoagulability in anaemic samples. 

Sampling site and technique did not have a significant influence on the change in coagulation status, neither did the thrombocyte number, despite some dogs having very low platelet numbers. An increase in haematocrit was associated with a major decrease in the probability of a change in coagulation status. This indicates that anaemic patients may be more susceptible to the effect of time on coagulation status. Further investigations are needed especially in anaemic patient groups of different severities to verify this effect. Furthermore, the influence of haemolysis on results could not be investigated, as data regarding serum colour was missing in most clinically ill patients. Haemolysis is associated with a reduced clot firmness und hypocoagulability in TEG analysis [33]. 

Normocoagulable samples showed the most prominent changes over time, while hypocoagulable samples were still affected, but less. This may be explained by the trend towards hypocoagulability over time, as no difference between mild and severe hypocoagulability was made. Due to the small sample size, this finding must be interpreted with caution. Furthermore, there is currently no standardized method to define normo-, hyper-, or hypocoagulability. Prolonged CT, reduced α, or reduced MCF can be indicative for hypocoagulability and a decreased time to clot formation, an increased α-angle or an increased MCF are indicative for hypercoagulability. We decided to use G values at T 30 for definition of normo-, hyper-, and hypocoagulability as G has been used in previous studies. G is a measure for the global clot strength and by representing an exponential transformation of MCF, it is more sensitive to haemostatic changes [26,35,36]. 

Another explanation for the change in ROTEM parameters and coagulation status over time may be the lack of duplicate measurements. The use of a high tissue factor concentration should lead to adequate activation of coagulation and results in a stable assay and should be less sensitive for pre-analytical influences [14]. Due to the serial measurement and limitation to 8 ROTEM channels, only a few samples were measured in duplicate. The coefficient of variation (CV) of these as well as additional samples have been evaluated in the concurrent study investigating the intra-assay and intra- and inter-operator variance [24]. Ex-TEM and Fib-TEM parameters showed only moderate repeatability while In-TEM parameters showed an excellent repeatability (CV < 10%). Clotting time and CFT showed the highest CV in all tests while MCF and G showed excellent repeatability in all tests, including Ex-TEM. While we cannot exclude an influence of this high variability on the results over time, our results suggest that there are significant changes and a trend towards hypocoagulable samples over time. 

Given the important change in coagulation status, time of analysis after blood sampling needs to be considered when interpreting ROTEM results in clinical patients. As long as the pathophysiology and cause of the change over time is not determined, it is not clear at which timepoint after blood sampling the “true” coagulation status (T10 vs. T30 vs. T70) is measured and reference intervals for all timepoints are required for appropriate interpretation of results. According to the results of our study, not only should institutional reference intervals be established, but the timing of analyses must also be standardized. The reference intervals presented in this study are only preliminary, as a larger patient population is required. 

The main limitation of the study is its small sample sizes of hypo- und hypercoagulable In-TEM and Fib-TEM tracings. Further studies to investigate the influence of time in dogs with abnormal coagulation status are of great interest. Another limitation is that for ill dogs, the sample handling was performed by different operators. While all operators were trained in ROTEM analysis, an influence by handler manipulation cannot be completely ruled out. 

## 5. Conclusions

In conclusion, ROTEM S tracings of canine citrated whole blood show a significant and clinically relevant change over time, being most prominent in Ex-TEM S tracings. Our results indicate that some parameters and tracings may become hypocoagulable over time. Specific reference intervals for different timepoints are required to avoid any false interpretation of a patient’s coagulation status. 

## Figures and Tables

**Table 1:** Ex-TEM, In-TEM, and Fib-TEM parameters over time.

Parameter			Time after Blood Sampling						Friedman
			**T10**		**T30**		**T70**		overall
Ex-TEM	n	reference range	median	range	median	range	median	range	*p*
CT	68	23–87	35	19–3600	36	20–656	34	20–544	0.260
CFT	68	85–357	219	48–3600	239	48–3600	258	66–3600	**<0.001**
alpha angle	68	42–77	53	1–84	52	1–85	49	1–85	**<0.001**
A10	68	21–55	34	1–66	32	1–68	31	4–62	**<0.001**
MCF	68	32–65	45	1–72	43	4–73	41	7–68	**<0.001**
ML	68	0–12	5	0–12	4	0–31	3	0–11	**<0.001**
MCE	68	45–142	82	1–256	75	4–272	69	7–210	**<0.001**
G	68	2253–5928	4077	51–12,801	3730	221–13,623	3445	357–10,485	**<0.001**
In-TEM	n	reference range	median	range	median	range	median	range	*p*
CT	35	133–210	181	115–239	185	136–230	184	133–240	0.188
CFT	35	59–201	94	39–236	107	33–437	133	38–277	**<0.001**
alpha angle	35	58–78	73	52–82	70	37–83	67	44–82	**0.001**
A10	35	35–61	46	33–69	46	24–73	42	31–70	**<0.001**
MCF	35	52–71	60	50–75	58	42–78	56	46–76	**<0.001**
ML	35	0–3	0	0–1	0	0–2	0	0–1	0.558
MCE	35	108–242	148	100–296	138	72–357	127	85–309	**<0.001**
G	35	5417–12,119	7414	5000–14,795	6902	3621–17,871	6364	4259–15,450	**0.001**
Fib-TEM	n	reference range	median	range	median	range	median	range	*p*
CT	28	21–112	36	26–2727	35	26–3600	38	24–3600	0.742
A10	28	2–9	5	2–21	5	1–24	4	1–20	**0.005**
MCF	28	2–9	5	2–22	6	1–23	4	1–21	**0.035**
ML	28	1–99	14	0–45	14	0–52	9	0–48	0.695
MCE	28	2–10	6	3–28	6	1–30	5	1–26	**0.039**
G	28	113–509	272	125–1382	291	51–1521	224	51–1319	0.286

CT—clotting time;CFT—clot formation time; ML—maximum lysis; MCF—maximum clot formation; MCE—maximum clot elasticity; A10—amplitude at 10 min; G—measure of clot strength; Ex-TEM—tissue factor activated temogram; In-TEM— allegic acid-activated temogram; Fib-TEM— tissue factor-activated temogram with platelet inhibition; T10—10 min after blood sampling; T30—30 min after blood sampling; T70—70–80 min after blood sampling. Significant *p*-values are presented in bold.

**Table 2:** Normocoagulable tracings of Ex-TEM, In-TEM, and Fib-TEM over time.

Parameter.			Time after Blood Sampling						Fried man	Wilcoxon		
			**T10**		**T30**		**T70**		overall	T10-30	T30-70	T10-70
Ex-TEM	n	reference range	median	range	median	range	median	range	*p*	*p*	*p*	*p*
CT	51	23–87	35	19–106	35	20–124	34	20–160	0.093	0.779	0.103	0.153
CFT	51	85–357	213	97–422	225	111–426	254	66–1215	**<0.001**	**0.004**	**0.004**	**<0.001**
alpha angle	51	42–77	53	41–83	53	41–82	49	27–85	**<0.001**	**0.134**	**<0.001**	**<0.001**
A10	51	21–55	35	24–54	33	23–46	31	15–62	**<0.001**	**<0.001**	**0.019**	**<0.001**
MCF	51	32–65	45	32–71	44	32–54	41	23–68	**<0.001**	**<0.001**	**0.028**	**<0.001**
ML	51	0–12	5	0–12	5	0–15	3	0–11	**<0.001**	**0.033**	**<0.001**	**<0.001**
MCE	51	45–142	82	48–249	78	48–115	70	30–210	**<0.001**	**0.001**	**0.024**	**<0.001**
G	51	2253–5928	4108	2392–12,464	3896	2396–5768	3519	1479–10,485	**<0.001**	**<0.001**	**0.024**	**<0.001**
In-TEM	n	reference range	median	range	median	range	median	range	*p*	*p*	*p*	*p*
CT	27	133–210	184	115–239	185	136–217	186	133–240	0.420	0.957	0.381	0.540
CFT	27	59–201	87	47–145	104	52–154	123	60–277	**<0.001**	0.101	**<0.001**	**<0.001**
alpha angle	27	58–78	73	63–80	70	62–79	67	44–78	**<0.001**	0.209	**0.001**	**<0.001**
A10	27	35–61	48	41–63	46	40–60	42	34–60	**<0.001**	0.241	**<0.001**	**<0.001**
MCF	27	52–71	60	54–71	59	53–70	56	47–72	**<0.001**	0.173	**<0.001**	**<0.001**
ML	27	0–3	0	0–1	0	0–2	0	0–1	0.558	0.480	0.739	0.257
MCE	27	108–242	150	117–240	145	112–239	127	88–257	**<0.001**	0.273	**<0.001**	**<0.001**
G	27	5417–12,119	7500	5868–11,981	7275	5620–11,932	6364	4377–12,831	**<0.001**	0.368	**<0.001**	**<0.001**
Fib-TEM	n	reference range	median	range	median	range	median	range	*p*	*p*	*p*	*p*
CT	23	21–112	36	26–159	36	26–841	38	24–241	0.786	0.793	0.592	0.637
A10	23	2–9	5	3–10	5	2–9	4	2–19	**0.018**	0.210	0.146	**0.045**
MCF	23	2–9	5	3–10	5	3–9	4	3–19	**0.043**	0.330	0.127	**0.049**
ML	23	1–99	14	1–43	15	4–52	10	0–48	0.393	0.420	0.144	0.648
MCE	23	2–10	5	3–11	6	3–10	5	3–24	**0.046**	0.307	0.245	**0.041**
G	23	113–509	266	143–572	266	129–494	221	145–1202	0.337	0.465	0.153	0.057

CT—clotting time; CFT—clot formation time; ML—maximum lysis; MCF—maximum clot formation; MCE—maximum clot elasticity; A10—amplitude at 10 min; G—measure of clot strength; Ex-TEM—tissue factor activated temogram; In-TEM— allegic acid-activated temogram; Fib-TEM— tissue factor-activated temogram with platelet inhibition; T10—10 min after blood sampling; T30—30 min after blood sampling; T70—70–80 min after blood sampling. Significant *p*-values are presented in bold.

**Table 3:** Hypocoagulable Ex-TEM, In-TEM, and Fib-TEM tracings over time.

Parameter			Time after Blood Sampling						Friedman	Wilcoxon		
			**T10**		**T30**		**T70**		overall	T10-30	T30-70	T10-70
Ex-TEM	n	reference range	median	range	median	range	median	range	*p*	*p*	*p*	*p*
CT	11	23–87	78	26–3600	118	24–656	69	31–544	0.850	0.505	0.646	0.790
A10	11	21–55	18	1–38	17	1–21	16	4–29	0.061	**0.013**	0.918	0.085
CFT	11	85–357	807	218–3600	891	567–3600	1171	281–3600	0.132	**0.022**	0.594	0.059
MCF	11	32–65	27	1–47	25	4–29	23	7–41	0.303	**0.036**	0.411	0.213
alpha angle	11	42–77	37	1–67	29	1–77	27	1–76	0.082	0.262	0.330	0.059
ML	11	0–12	1	0–12	1	0–10	2	0–9	**0.044**	0.084	0.221	0.056
MCE	11	45–142	36	1–89	33	4–42	30	7–68	0.319	**0.032**	0.655	0.155
G	11	2253–5928	1804	51–4459	1641	221–2091	1493	357–3057	0.336	**0.026**	0.594	0.182
In-TEM	n	reference range	median	range	median	range	median	range	*p*	*p*	*p*	*p*
CT	6	133–210	179	132–229	212	174–230	194	177–211	0.311	0.116	0.463	0.249
A10	6	35–61	38	33–41	36	24–37	35	31–41	0.554	0.138	0.833	0.345
CFT	6	59–201	165	141–236	190	169–437	207	130–253	0.438	0.225	0.600	0.463
MCF	6	52–71	53	50–56	52	42–52	52	46–57	0.337	**0.042**	0.674	0.527
alpha angle	6	58–78	62	52–65	57	37–61	58	50–66	0.513	0.141	0.463	0.599
ML	6	0–3	0	0	0	0	0	0		1.000	1.000	1.000
MCE	6	108–242	111	100–126	108	72–108	108	85–133	0.337	**0.043**	0.528	0.528
G	6	5417–12,119	5527	5000–6324	5379	3621–5417	5421	4259–6646	0.337	**0.043**	0.600	0.528
Fib-TEM	n	reference range	median	range	median	range	median	range	*p*	*p*	*p*	*p*
CT	2	21–112	1424	121–2727	3600	3600–3600	3314	3027–3600	0.156	0.180	0.317	0.180
A10	2	2–9	2	2–2	1	1–1	1	1–1	0.135	0.157	1.000	0.157
MCF	2	2–9	3	2–3	1	1–1	2	1–2	0.156	0.180	0.317	0.157
ML	2	1–99	39	32–45	0	0	3	0–6	0.156	0.180	0.317	0.180
MCE	2	2–10	3	3–3	1	1–1	2	1–2	0.156	0.157	0.317	0.180
G	2	113–509	135	125–145	51	51–51	78	51–106	0.156	0.180	0.317	0.180

CT—clotting tim; CFT—clot formation time; ML—maximum lysis; MCF—maximum clot formation; MCE—maximum clot elasticity; A10—amplitude at 10 min; G—measure of clot strength; Ex-TEM—tissue factor activated temogram; In-TEM— allegic acid-activated temogram; Fib-TEM— tissue factor-activated temogram with platelet inhibition; T10—10 min after blood sampling; T30—30 min after blood sampling; T70—70–80 min after blood sampling. Significant *p*-values are presented in bold.

**Table 4:** Hypercoagulable Ex-TEM, In-TEM, and Fib-TEM tracings over time.

Parameter			Time after Blood Sampling						Friedman	Wilcoxon		
			**T10**		**T30**		**T70**		overall	T10-30	T30-70	T10-70
Ex-TEM	n	reference range	median	range	median	range	median	range	*p*	*p*	*p*	*p*
CT	6	23–87	28	25–96	28	25–35	28	20–29	0.953	0.463	0.684	1.000
A10	6	21–55	52	41–66	53	45–68	48	44–49	0.154	0.343	0.058	0.138
CFT	6	85–357	112	48–138	97	48–146	119	106–155	**0.032**	0.686	**0.028**	0.093
MCF	6	32–65	60	50–72	62	56–73	58	55–60	0.108	0.216	0.058	0.138
alpha angle	6	42–77	74	66–84	77	62–85	71	61–84	0.154	0.527	0.115	0.138
ML	5	0–12	2	2–7	2	2–6	2	0–6	0.097	0.317	0.180	0.102
MCE	6	45–142	148	100–256	160	125–272	137	120–147	0.084	0.225	**0.046**	0.116
G	6	2253–5928	7390	4985–12,801	7995	6231–13,623	6837	6022–7368	0.084	0.225	**0.046**	0.116

CT—clotting time; CFT—clot formation time; ML—maximum lysis; MCF—maximum clot formation; MCE—maximum clot elasticity; A10—amplitude at 10 min; G—measure of clot strength; Ex-TEM—tissue factor activated temogram; T10—10 min after blood sampling; T30—30 min after blood sampling; T70—70–80 min after blood sampling. Significant *p*-values are presented in bold.

**Table 5:** Coagulation status over time.

TEST	Coagulation Status	T10	T30	T70	*p*
		n/N (%)	n/N (%)	n/N (%)	
Ex-tem	normocoagulable	52/70 (74%)	53/70 (75%)	50/68 (73%)	**<0.001**
	hypocoagulable	6/70 (9%)	11/70 (16%)	12/68 (18%)	0.336
	hypercoagulable	12/70 (17%)	6/70 (9%)	6/68 (9%)	0.084
In-tem	normocoagulable	32/35 (91%)	27/35 (77%)	28/35 (80%)	**<0.001**
	hypocoagulable	2/35 (6%)	6/35 (17%)	5/35 (14%)	**0.043**
	hypercoagulable	1/35 (3%)	2/35 (6%)	2/35 (6%)	0.135
Fib-tem	normocoagulable	25/28 (89%)	23/28 (82%)	22/28 (79%)	0.337
	hypocoagulable	0/28 (0%)	2/28 (7%)	2/28 (7%)	0.156
	hypercoagulable	3/28 (11%)	3/28 (11%)	4/28 (14%)	0.097

**Table 6:** Reference intervals for selected parameters of Ex-TEM S, In-TEM S, and Fib-TEM S 10, 30, and 70 min after blood sampling.

Test	Parameter	Time after Sampling	n	Reference Interval (RI by Jud)	Lower and Upper 90% CI	Friedmann Test
Ex-tem	CT (s)	10 min	36	26–88	NA	0.384
		30 min	36	23–113 (23–87)	NA	
		70 min	36	25–140	NA	
	CFT (s)	10 min	36	96–327	78–125 and 299–355	<0.00001
		30 min	36	76–408 (85–357)	36–117 and 367–449	
		70 min	33	128–482	NA	
	A (°)	10 min	36	39–73	35–43 and 68–77	<0.00001
		30 min	36	37–72 (42–77)	32–41 and 68–77	
		70 min	36	28–70	23–33 and 65–75	
	A10 (mm)	10 min	36	25–49	22–28 and 44–50	<0.00001
		30 min	36	21–46 (21–55)	17–24 and 43–49	
		70 min	36	17–46	14–21 and 42–49	
	MCF (mm)	10 min	36	36–58	33–38 and 54–59	<0.0001
		30 min	36	32–56 (32–65)	29–35 and 53–60	
		70 min	36	28–56	24–31 and 53–60	
	MCE	10 min	36	49–127	40–58 and 118–137	<0.0001
		30 min	36	50–141 (45–142)	NA	
		70 min	36	31–120	20–42 and 109–131	
	G	10 min	36	2742–6965	NA	<0.0001
		30 min	36	2509–7023 (2253–5928)	NA	
		70 min	36	1535–5996	989–2080 and 5450–6541	
	ML (%)	10 min	36	1–9		<0.0001
		30 min	35	0–15 (0–12)		
		70 min	36	0–8	0 and 7–9	
In-tem	CT (s)	10 min	25	126–234	110–142 and 218–250	0.20061
		30 min	25	140–233 (133–210)	126–154 and 220–247	
		70 min	25	136–240	120–151 and 225–256	
	CFT (s)	10 min	25	53–234	NA	<0.0001
		30 min	24	27–207 (59–201)	0–54 and 180–234	
		70 min	25	36–246	3–68 and 224–289	
	A (°)	10 min	25	55–82	51–60 and 78–86	0.015
		30 min	25	39–79 (58–78)	NA	
		70 min	25	49–80		
	A10 (mm)	10 min	25	31–61	29–36 and 57–66	<0.0001
		30 min	25	27–63 (35–61)	22–33 and 58–68	
		70 min	25	49–80	45–54 and 76–85	
	MCF (mm)	10 min	25	50–85	NA	0.001
		30 min	25	45–72 (52–71)	41–49 and 68–75	
		70 min	24	46–68	NA	
	MCE	10 min	24	100–230	NA	0.003
		30 min	25	76–237 (108–242)	NA	
		70 min	24	86–207	NA	
	G	10 min	24	5020–11,508	NA	0.006
		30 min	25	3781–11,834 (5417–12,119)	NA	
		70 min	24	4289–10,371	NA	
	ML (%)	10 min	25	0–1	NA	0.696
		30 min	25	0–1 (0–3)	NA	
		70 min	25	0–1	NA	
Fib-tem	CT (s)	10 min	21	28–158	NA	0.734
		30 min	19	27–92 (21–112)	NA	
		70 min				
	A10 (mm)	10 min	22	0–10	0–2 and 8–11	0.013
		30 min	22	0–10 (2–9)	0–2 and 8–11	
		70 min	22	0–13	0–4 and 4–17	
	MCF (mm)	10 min	20	3–10	NA	0.074
		30 min	20	2–9 (2–9)	0–3 and 8–11	
		70 min	20	3–19	NA	
	MCE	10 min	20	3–11	NA	0.067
		30 min	20	3–12 (2–10)	NA	
		70 min	20	3–24	NA	
	G	10 min	20	143–572	NA	0.544
		30 min	20	62–523 (113–509)	0–137 and 446–600	
		70 min	19	145–561	NA	

NA—not available; CT—clotting time; CFT—clot formation time; ML—maximum lysis; MCF—maximum clot formation; MCE—maximum clot elasticity; A10—amplitude at 10 min; G—measure of clot strength; Ex-TEM—tissue factor activated temogram; In-TEM—allegic acid-activated temogram; Fib-TEM— tissue factor-activated temogram with platelet inhibition.

## Data Availability

Data are available on request.
